# Overcoming species barriers: an outbreak of *Lagovirus europaeus* GI.2/RHDV2 in an isolated population of mountain hares (*Lepus timidus*)

**DOI:** 10.1186/s12917-018-1694-7

**Published:** 2018-11-26

**Authors:** Aleksija S. Neimanis, Harri Ahola, Ulrika Larsson Pettersson, Ana M. Lopes, Joana Abrantes, Siamak Zohari, Pedro J. Esteves, Dolores Gavier-Widén

**Affiliations:** 1 Department of Pathology and Wildlife Diseases, National Veterinary Institute (SVA), 751 89 Uppsala, Sweden; 2 Department of Biomedical Sciences and Veterinary Public Health, Swedish University of Agricultural Sciences (SLU), Box 7028, 750 07 Uppsala, Sweden; 3 Department of Microbiology, National Veterinary Institute (SVA), 751 89 Uppsala, Sweden; 4 CIBIO/InBio, Centro de Investigação em Biodiversidade e Recursos Genéticos, Universidade do Porto, Campus Agrário de Vairão, 4485-661 Vairão, Portugal; 5 Department of Anatomy and Unit for Multidisciplinary Research in Biomedicine (UMIB), Institute of Biomedical Sciences Abel Salazar (ICBAS), University of Porto, Porto, Portugal; 6 Departamento de Biologia, Faculdade de Ciências, Universidade do Porto, R. Campo Alegre s/n, 4169-007 Porto, Portugal; 7 Instituto de Investigação e Formação Avançada em Ciências e Tecnologias da Saúde (CESPU), Gandra, Portugal

**Keywords:** Rabbit hemorrhagic disease, *Lagovirus europaeus* GI.2, RHDV2, *Lepus timidus*, Hare, Virus, Wildlife, Hepatitis

## Abstract

**Background:**

Prior to 2010, the lagoviruses that cause rabbit hemorrhagic disease (RHD) in European rabbits (*Oryctolagus cuniculus*) and European brown hare syndrome (EBHS) in hares (*Lepus* spp*.*) were generally genus-specific. However, in 2010, rabbit hemorrhagic disease virus 2 (RHDV2), also known as *Lagovirus europaeus* GI.2, emerged and had the distinguishing ability to cause disease in both rabbits and certain hare species. The mountain hare (*Lepus timidus*) is native to Sweden and is susceptible to European brown hare syndrome virus (EBHSV), also called *Lagovirus europaeus* GII.1. While most mountain hare populations are found on the mainland, isolated populations also exist on islands. Here we investigate a mortality event in mountain hares on the small island of Hallands Väderö where other leporid species, including rabbits, are absent.

**Results:**

Post-mortem and microscopic examination of three mountain hare carcasses collected from early November 2016 to mid-March 2017 revealed acute hepatic necrosis consistent with pathogenic lagovirus infection. Using immunohistochemistry, lagoviral capsid antigen was visualized within lesions, both in hepatocytes and macrophages. Genotyping and immunotyping of the virus independently confirmed infection with *L. europaeus* GI.2, not GII.1. Phylogenetic analyses of the *vp60* gene grouped mountain hare strains together with a rabbit strain from an outbreak of GI.2 in July 2016, collected approximately 50 km away on the mainland.

**Conclusions:**

This is the first documented infection of GI.2 in mountain hares and further expands the host range of GI.2. Lesions and tissue distribution mimic those of GII.1 in mountain hares. The virus was most likely initially introduced from a concurrent, large-scale GI.2 outbreak in rabbits on the adjacent mainland, providing another example of how readily this virus can spread. The mortality event in mountain hares lasted for at least 4.5 months in the absence of rabbits, which would have required virus circulation among mountain hares, environmental persistence and/or multiple introductions. This marks the fourth *Lepus* species that can succumb to GI.2 infection, suggesting that susceptibility to GI.2 may be common in *Lepus* species. Measures to minimize the spread of GI.2 to vulnerable *Lepus* populations therefore are prudent.

**Electronic supplementary material:**

The online version of this article (10.1186/s12917-018-1694-7) contains supplementary material, which is available to authorized users.

## Background

Pathogenic lagoviruses (Family *Caliciviridae*) cause hepatitis in leporids. The disease is referred to as rabbit hemorrhagic disease (RHD) or European brown hare syndrome (EBHS) depending on whether it affects rabbits or hares. Viruses identified prior to 2010 are generally considered to be genus-specific and do not cause clinical disease in animals less than five weeks of age. Disease caused by rabbit hemorrhagic disease virus (RHDV) and its antigenic variant (RHDVa), also collectively known as *Lagovirus europaeus* GI.1 [1], is almost exclusively confined to domestic and wild European rabbits (*Oryctolagus cuniculus*). Similarly, European brown hares (*Lepus europaeus*) and other hare species including mountain hares (*Lepus timidus*) are susceptible to EBHS virus (EBHSV) [2], also called *Lagovirus europaeus* GII.1 [1], but not to GI.1 viruses. However, in 2010, RHDV2 or RHDVb, also known as *L. europaeus* GI.2 [1], emerged in France [3]. Differing significantly from the previously detected viruses, GI.2 has a broader age and host range. GI.2 can cause clinical disease and death in rabbits as young as 11 days old [4]. In addition to infecting rabbits, GI.2 can also cause EBHS-like disease in the Sardinian Cape hare (*Lepus capensis mediterraneus*) [5], the Italian hare (*Lepus corsicanus*) [6] and the European brown hare [7–9].

The mountain hare (*Lepus timidus*) is an arctic/subarctic species that inhabits the tundra and taiga habitats of northern Europe and Asia [10]. While listed as a species of least concern globally [11], certain regions in Europe and Russia have been experiencing gradual population declines [12]. This species is particularly prone to dramatic population crashes on islands [13]. In Sweden, the mountain hare is a native species and it historically ranged throughout the country. Isolated populations of mountain hares also exist on many small Swedish islands because of relatively recent introductions [10]. While populations in the north are robust, mountain hare range and density have decreased in south and central Sweden since the beginning of the twentieth century, and mountain hares have completely disappeared from the far south [12]. Postulated reasons for the decline include negative impacts from introduced European brown hares (*Lepus europaeus*), predation pressure, competition with other herbivores, or landscape change [12]. While competitive exclusion by, and hybridization with, brown hares are considered primary candidates for the decline of southern mountain hare populations in Sweden, disease-mediated competition through, for example, GII.1 or *Francisella tularensis* may also play a role [12]. Although GII.1 can infect and cause disease in both European brown hares and mountain hares, documented cases of EBHS in Sweden are geographically restricted to the range of European brown hares. Confirmed cases in mountain hares have only occurred in the southern half of the country where the ranges of European brown and mountain hares overlap [14, 15]. This suggests that the virus circulates mainly in European brown hares and that mountain hares may be spillover hosts [14].

In the early autumn of 2016, managers of a small island nature reserve off the Swedish west coast began to find carcasses of mountain hares. Here we describe an outbreak of a pathogenic lagovirus in this island population of mountain hares and provide evidence for the further expansion of GI.2 host range. The outbreak occurred on an island without rabbits or European brown hares and we explore the epidemiological circumstances of the outbreak, describe the pathology in mountain hares, investigate the molecular epidemiology of GI.2, and discuss the implications of disease introductions for isolated wildlife populations.

## Methods

### Study site

**Fig. 1:**
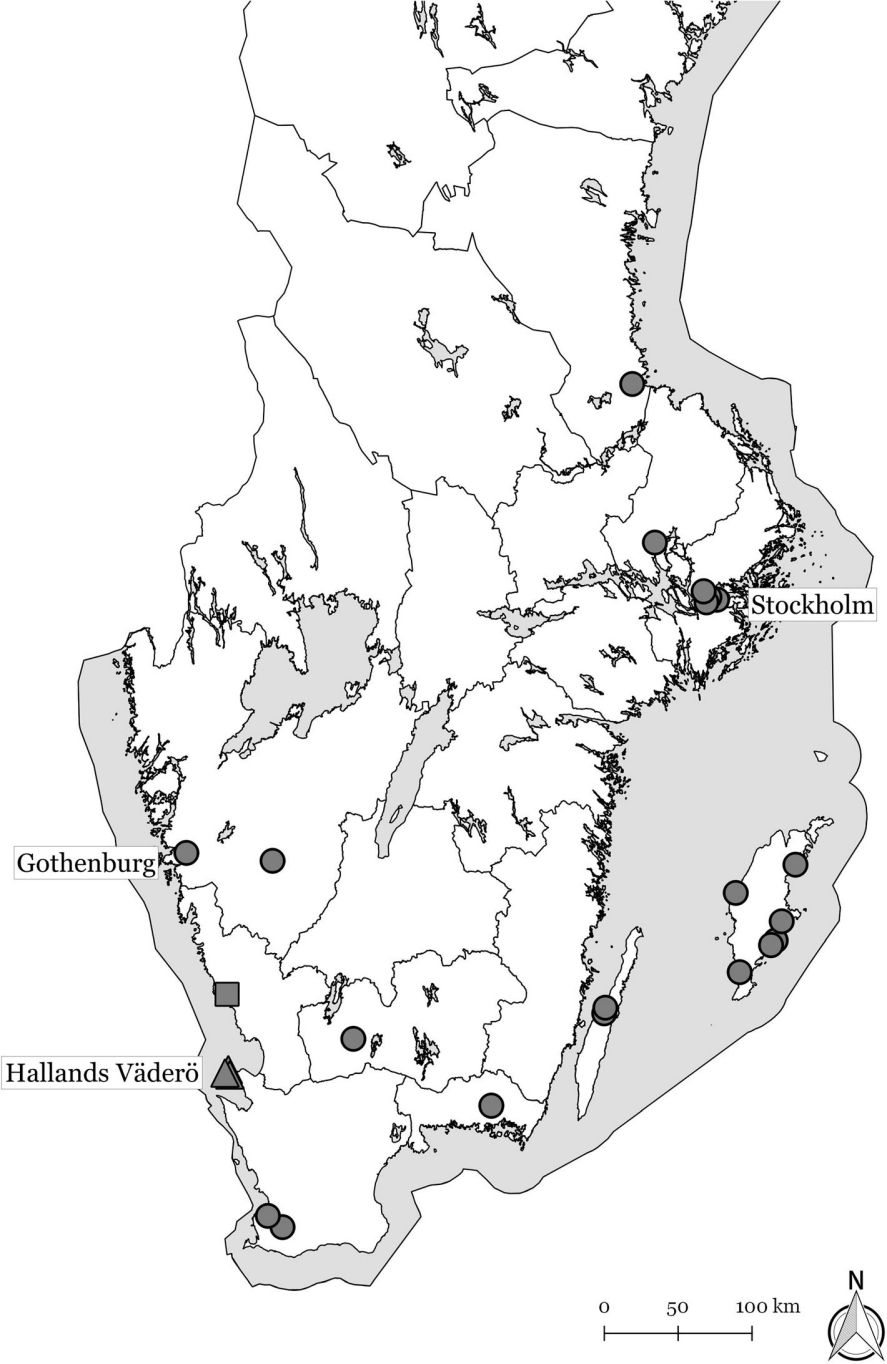
A map of the southern Sweden depicting cases of *Lagovirus europaeus* G1.2/RHDV2/b infection from which the *vp60* gene sequences originated. The three mountain hares from Hallands Väderö (2147, 2381 and 1208) are represented by a triangle (▲), the rabbit from Falkenberg (1251) is depicted by a square (■) and other rabbits are shown with a circle (●). The base map is provided by Lantmäteriet, Sweden under the open data license CC0

Hallands Väderö (N 56° 26.6′, E 12° 33.7′) is a small, 310 ha island 3 km off the southwest coast of Sweden, accessible only by boat (Fig. 1). It is a nature reserve with no permanent residents living on the island, but is host to approximately 50,000 visitors per year, primarily in the summer months. The island is also home to an introduced, managed mountain hare population and organized hunts are typically held 1–2 times per year. The earliest documented introduction of hares occurred in 1857 from Godska Sandön, a Swedish island in the Baltic Sea, and numerous restocking events have occurred since then [16]. There are no European brown hares or wild rabbits on the island, making the mountain hare the only leporid species present.

### Animals

In September 2016, managers of Hallands Väderö began finding mountain hare carcasses. Remains of approximately 15 animals were noted from September to December 2016. In the beginning of November 2016, the first dead hare (animal 2147) was collected, frozen and transported to the National Veterinary Institute (SVA), Uppsala, Sweden for necropsy on November 17. Two other dead hares found at the beginning of December 2016 (animal 2381) and middle of March 2017 (animal 1208) were frozen and transported to SVA for examination on December 19, 2016 and March 29, 2017, respectively (Table 1).

**Table 1:** Mountain hares (*Lepus timidus*) found dead or hunted on Hallands Väderö, Sweden

Hare identification number	Found dead (D) or hunted (H)	Date collected	Sex^a^	Age^b^	Body condition
2147	D	November 2016	F	Y	Poor
2381	D	December 2016	F	A	Normal
1208	D	March 2017	M	U	Emaciated
123	H	January 14, 2017	M	NE	Normal
124	H	January 14, 2017	M	NE	Normal
126	H	January 14, 2017	M	NE	Normal
127	H	January 14, 2017	F	A	Normal
2539	H	September 21, 2017	NE	NE	Normal
2540	H	September 21, 2017	NE	NE	Normal

^a^*F* female, *M* male, *NE* not examined

^b^*Y* young of year, *A* adult, *U* unable to determine *NE*, not examined

**Fig. 2:**
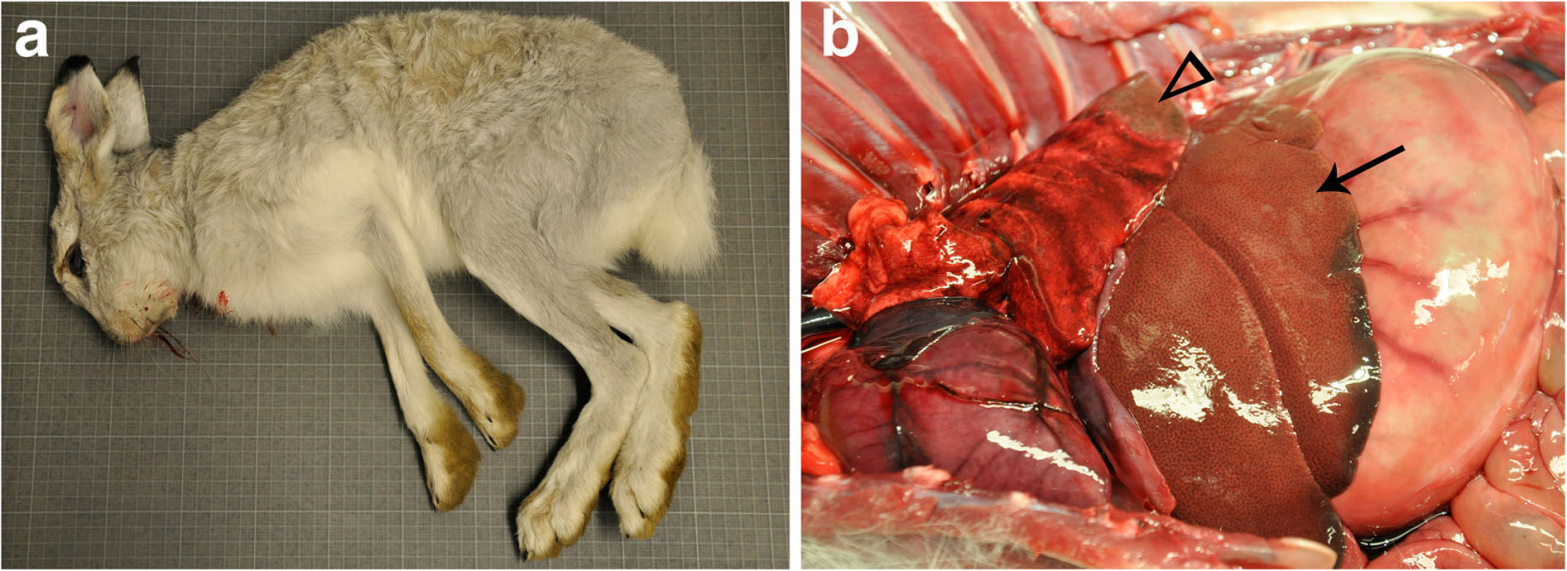
A mountain hare (*Lepus timidus*) that died of Lagovirus europaeus GI.2/RHDV2/b infection on the island of Hallands Vädero, Sweden. **a** Characteristic external features include a grey-white winter pelage. **b** Post-mortem findings. The liver shows pallor and enhanced reticular pattern (arrow). Distal lung lobes are consolidated because of nematode parasitism (arrowhead)

All three animals showed characteristic features of *Lepus timidus*. Compared to *Lepus europaeus*, they were smaller, had shorter ears and front limbs and the forehead was rounder [10]. The pelage was in varying stages of moulting from grey-brown to grey-white (Fig. 2). The animal found in November had the greatest proportion of brown in its pelage and the animal in March was almost completely grey-white. To confirm species identity, molecular analyses were also carried out on liver samples of two of the hares (2147 and 1208). DNA was extracted with the EasySpin Genomic DNA Minipreps Tissue Kit (Citomed, Portugal) following manufacturer’s instructions. When considered together, the nuclear genes uncoupling protein 2 (*upc2*) and immunoglobulin heavy constant gamma (*ighg*) and the mitochondrial gene cytochrome b (*cytb*) distinguish *L. timidus* from the other leporid species. Amplification of these markers was carried out using the primers described in [17, 18]; PCR conditions are available upon request. After purification, PCR products were sequenced on an automatic sequencer ABI PRISM 310 Genetic Analyzer (PE Applied Biosystems, USA) using the amplification primers. Sequences were submitted to the publicly available GenBank database (https://www.ncbi.nlm.nih.gov/genbank) under the accession numbers MH107277–82. Genetic distances between the sequences of 2147 and 1208 and other lagomorphs were calculated for each gene in MEGA6 [19] using the following parameters: p-distance method, 500 replicates, pairwise deletion.

Management of the mountain hare population on Hallands Väderö included a hunt in January and September 2017. Sampling of these hunted animals provided an opportunity to screen for evidence of GI.2 infection in these apparently healthy animals during (*n* = 4, January) and after (*n* = 2, September) the outbreak. Fresh liver and duodenum samples were collected from animals hunted in January 2017 and frozen at − 20 °C. Formalin-fixed tissues from these animals included liver, spleen, duodenum, jejunum, ileum, sacculus rotundus and cecal appendix. In September 2017, fresh and formalin-fixed liver were collected.

### Post-mortem and microscopic examination and bacteriology

All three fallen animals were examined by necropsy. Nutritional condition was determined according to the following criteria: emaciated (visible muscle atrophy, absence of visible fat stores), poor (scant to no visible internal fat stores, but no visible muscle atrophy), normal (at a minimum, fat stores visible in mesentery and around kidneys). A suite of internal organs including liver, spleen, lung, kidney, bone marrow, brain, small intestine, colon, heart, trachea, salivary gland, nasal mucosa and skin were fixed in 10% neutrally buffered formalin for microscopic examination. Not all tissues were collected from all animals. Liver was frozen at − 20 °C and − 70 °C for additional analyses.

Formalin-fixed tissues from the found dead and hunted hares were processed and embedded in paraffin for microscopic examination. Sections 3 to 4 μm thick were stained with Mayer’s hematoxylin and eosin [20]. Liver sections also were stained with von Kossa to visualize calcium deposits [21].

Even though no organisms were seen microscopically, the mild to moderate heterophilic infiltration observed in the liver of 2147 prompted the submission of liver from this animal to the Department of Microbiology, National Veterinary Institute, Uppsala, Sweden for routine aerobic bacterial culture. Samples were inoculated onto blood agar plates containing 5% horse blood and bromocresol purple lactose agar plates and held at 37 °C under aerobic conditions. Plates were inspected for growth at 24 and 48 h after inoculation.

### Immunohistochemistry

Immunohistochemistry was used to detect the presence of lagovirus antigen in the formalin-fixed and paraffin-embedded tissues using a panlagovirus antibody cocktail of 3H6 + 6G2 IgG1 mouse monoclonal antibodies [22] kindly provided by the OIE Reference Laboratory for RHD, Brescia, Italy. Immunohistochemical staining was performed on liver, spleen, lung, bone marrow and kidney from the three fallen hares and on the liver and small intestine of hares hunted in January. Methods, antibodies and reagents are described in detail in Neimanis et al. [23] with the following modifications. After treatment with hydrogen peroxide, sections were treated with proteinase K (Dako Proteinase K, Agilent Technologies Sweden AB, Kista, Sweden) for six minutes to demask antigens. Visualization of bound antibodies was facilitated with either the chromagen diaminobenzidine (DAB) (Dako Liquid DAB+ Substrate Chromagen System, Agilent Technologies Sweden AB, Kista, Sweden) or the chromagen 3-amino-9-ethylcarbazole (AEC) (Dako AEC+ High Sensitivity Substrate Chromagen, Agilent Technologies Sweden AB, Kista, Sweden). The liver from a rabbit confirmed to be infected with GI.2 by PCR, sequencing and immunological characterization [24] and/or the liver from a European brown hare confirmed to be infected with GII.1 by nested PCR [25] was used as the positive tissue control while the liver from a healthy rabbit and/or European brown hare (negative for lagoviruses by PCR) served as the negative tissue control.

### Immunological virus typing

Liver samples from all three fallen hares were submitted to the OIE Reference Laboratory for RHD in Brescia, Italy for immunological typing of the lagovirus. Briefly, typing analysis was performed using the sandwich ELISA described in the OIE Terrestrial Manual for RHD (2016) [26]. Four pools of monoclonal antibodies specific for GI.1b-d/RHDV [22], GI.1a/RHDVa [27], GI.2/RHDV2 [5] and GII.1/EBHSV [15] were used. Samples (10% liver homogenates) were tested at the dilution 1/5 and 1/30 and classified as positive when the OD of the sample exceeded that of the negative control by at least 0.15 at dilution 1/5. A more detailed description of the methods is provided in Neimanis et al. [24].

### Genotyping and phylogenetic analyses

Based on gross and microscopic findings typical for pathogenic lagovirus infection in the three hares found dead, various molecular analyses were carried out on liver samples to confirm the presence of a lagovirus and to genotype the virus. For hares 1208 and 2147, samples were first analyzed for the presence of GI and GII.1 with two nested PCRs using primers and methods as described in Bascuñana et al. [25]. In both cases, GI but not GII.1 RNA was detected. Because this nested PCR does not differentiate between GI.1 and GI.2 viruses [24], liver samples from these two hares and from 2381 were analyzed by GI.2-specific RT-qPCR as described in Neimanis et al. [24]. To screen for the presence of GI.2, liver samples from all six hunted hares were also analyzed by this same GI.2-specific RT-qPCR.

For samples found to contain GI.2 RNA by RT-qPCR, sequencing of the entire *vp60* gene following methods in Neimanis et al. [24] was carried out. In 2016 and 2017, a concurrent, widespread outbreak of GI.2 occurred in wild and domestic rabbits in the southern half of Sweden [24]. To gain insight into the origins of GI.2 in mountain hares on Hallands Väderö, the entire *vp60* gene was also sequenced from the livers of ten additional rabbits known to have died from GI.2 infection for phylogenetic comparison with hare strains. Rabbit samples were selected to represent the broad spatial and temporal range of the outbreak. Locations from where these 10 rabbits, the three fallen mountain hares and an additional 11 rabbits previously reported with GI.2 in Sweden [24] originated are presented in Fig. 1. All *vp60* gene sequences generated in this study were deposited in GenBank under accession numbers MH341501–13.

To identify homologous sequences in GenBank, a Basic Local Alignment Search Tool (BLAST, http://www.ncbi.nlm.nih.gov/BLAST/) was used on the *vp60* gene sequences from the three fallen hares. Additionally, evolutionary relationships between the mountain hare viruses and other lagoviruses were explored. Sequences were aligned using the Clustal W algorithm before building phylogenetic trees using MEGA6: Molecular Evolutionary Genetics Analysis [19]. The best-fit nucleotide substitution model (GTR + G + I) was determined with the same software. The final alignment included *vp60* gene sequences generated for this study (from the three mountain hares and 10 rabbits) and publicly available GI.2 sequences from Europe and Australia, including 11 previously published sequences from Sweden from 2013 to 2016 [24]. GI.1, GI.4 and GII.1 sequences, as well as MRCV (a lagovirus with variable pathogenicity [28]) and other sequences not assigned to a genotype, were included in the analysis, producing a final dataset of *n* = 364. A list of all sequences used is provided as supplementary material in Additional file 1. Trees were built using the Maximum likelihood (ML) method and statistical support for the nodes was estimated using 2000 bootstrap replicates.

## Results

Sex, age and nutritional condition of the mountain hares are presented in Table 1. Body condition of the fallen hares ranged from normal to emaciated whereas the hunted hares were all in normal nutritional condition. Age was not assessed for the majority of the hunted animals and sex was not determined for animals hunted in September 2017.

### Hares found dead

#### Molecular species identification

The obtained sequences for *ucp2*, *ighg*-hinge and *cytb* were visually inspected and aligned with publicly available sequences for lagomorphs. Only species with information for at least two markers were considered. Genetic distances indicate that our samples are closest to three hare species *L. timidus*, *L. arcticus* and *L. othus* and clearly distinct from the European brown hare. Despite the lack of resolution, we confirm the species as being *L. timidus*, which is consistent with the morphological characterization and species geographic range (*L. arcticus* inhabits the coastal regions of Greenland, northern Quebec, northern Manitoba, Arctic islands and western Newfoundland; *L. othus* ranges from west to southwest Alaska). The three arctic hare species (*L. timidus*, *L. arcticus* and *L. othus*) are closely related and their status as independent species has been critically discussed [29, 30].

#### Gross pathology

All three animals found dead and examined by necropsy had subtle to moderate liver lesions in which the liver was mildly to moderately discoloured (light brown to orange-pink), had an accentuated lobular pattern in the parenchyma, and was friable (Fig. 2). All three animals also displayed mild to moderate pulmonary congestion and multifocal to coalescing hemorrhage and/or edema. There was mild to moderate splenomegaly in all animals and the spleen was discoloured dark red in hares 2147 and 2381. All animals had soft, unformed feces in the rectum and in 2381, the cecal appendix and sacculus rotundus were diffusely dark red. There was mild to moderate consolidation of the distal margins of the caudal lung lobes consistent with nematode infestation in all animals (Fig. 2) and few to more frequent 1 × 2 mm beige, oblong foci consistent with coccidial organisms were seen in the small intestine of hares 2147 and 1208.

#### Microscopic pathology

**Fig. 3:**
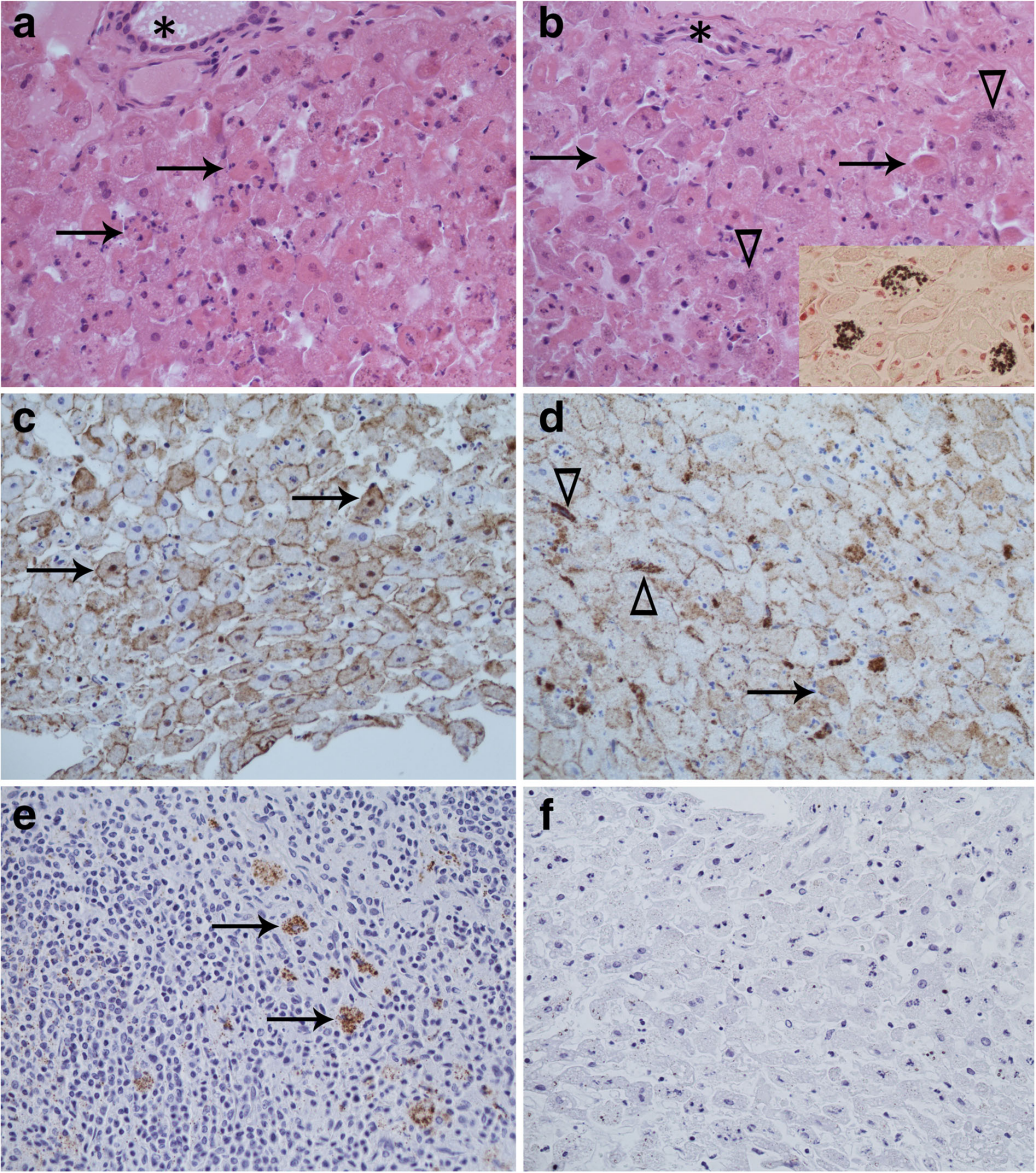
Microscopic lesions and viral antigen localization in mountain hares (*Lepus timidus*) that died of *Lagovirus europaeus* GI.2/RHDV2/b infection. **a** Acute hepatocellular apoptosis and necrosis. Dead cells are denoted with an arrow and they frequently are surrounded by heterophils. The portal region is denoted by an asterisk. **b** Acute hepatocellular apoptosis and necrosis (arrows). Arrowhead denote hepatocellular calcification and the asterisk marks the portal area. Insert: von Kossa histochemical stain with calcium granules stained black. **c** Immunohistochemical visualization of viral capsid antigen (brown) in the liver of hare 2147. Arrows denote hepatocytes with both intracytoplasmic and intranuclear staining. **d** Immunohistochemical visualization of viral capsid antigen (brown) in the liver of hare 1208. Intracytoplasmic staining of hepatocytes (arrow) and Kupffer cells (arrowhead). **e** Immunohistochemical visualization of viral capsid antigen (brown) in the spleen of hare 1208. Arrows indicate intracytoplasmic staining of macrophages in the red pulp. **f** Negative immunoglobulin control of the liver of 1208

All fallen hares had been previously frozen and were moderately to severely autolyzed. However, liver lesions could still be detected. All animals displayed acute necrosis and/or apoptosis of individual hepatocytes characterized by hypereosinophilic, hyaline cytoplasm, cell shrinkage, rounding up and dissociation from the hepatic plate (Fig. 3). Affected hepatocytes were observed throughout the lobule. In hares 2147 and 1208, small scattered, focal areas of lytic necrosis were also seen. Additionally, dark grey-basophilic, irregular intracytoplasmic granules confirmed to be mineral using von Kossa histochemical stain were observed in scattered (2147) to frequent (1208) hepatocytes (Fig. 3). In all hares, mildly to moderately increased numbers of inflammatory cells, most consistent with heterophils, were observed in sinusoids and occasionally surrounding individual hepatocytes (Fig. 3). There also was mild (2147 and 1208) to moderate (2381) microvesiculation of hepatocytes consistent with lipid. Hare 2381 had a mild trematode infestation evidenced by scattered trematode eggs observed within larger bile ducts.

Microscopic examination of the lungs and small intestine confirmed granulomatous pneumonia from lungworm infection and intestinal coccidiosis, respectively. Autolysis precluded further interpretation of other tissues.

#### Immunohistochemistry

Viral antigen was detected in the liver of all three fallen hares (Fig. 3). In 2147 and 2381, the antigen was primarily confined to hepatocytes as fine intracytoplasmic stippling or concentrated at the cell membrane, although occasional intranuclear staining also was seen (Fig. 3). In 1208, staining frequently was seen within the cytoplasm of both hepatocytes and Kupffer cells (Fig. 3), and very rarely within hepatocellular nuclei. Immunostaining was not detected in the negative controls.

Immunohistochemical evaluation of the spleen of 2147 and 2381 was hindered by autolysis and artifact. However, within the spleen of 1208, viral antigen was clearly and coarsely clumped within the cytoplasm of macrophages (Fig. 3). Poor tissue quality precluded interpretation of immunohistochemistry for remaining tissues.

#### Bacteriology

No bacterial infection was identified in the liver of hare 2147.

### Hunted hares

#### Pathology and immunohistochemistry

No significant lesions were observed grossly in the hunted hares. Microscopically, no lesions consistent with pathogenic lagoviruses were seen in the liver. In hares 126 and 127, hilar bile ducts contained cross-sections of adult trematodes and/or thick-walled yellow-brown trematode eggs that were accompanied by mild to moderate periductal eosinophilic and lymphoplasmacytic inflammation. Small intestine only was available for examination in hares hunted in January 2017. Although scattered intraluminal nematodes were observed in hares 124, 126 and 127, the intestinal tissue was unremarkable.

No viral antigen was detected in the liver or small intestine of hares hunted in January 2017 and immunohistochemistry was not performed on the two hares hunted in September 2017.

### Immunological virus typing

In all three fallen hares, the lagovirus in the liver was identified as GI.2 by the specific pool of MAbs directed against GI.2. Results for the other pools of MAbs specific for GI.1b-d, GI.1a and GII.1 were negative. Additionally, the high OD values registered at the dilution of 1/30 are consistent with acute lagovirus infection and cause of the death of the hares.

### Molecular detection and phylogenetic analysis

A 127-base pair fragment from the *vp60* capsid gene of GI.2 was amplified in large quantities in the liver of all three hares that were found dead. The threshold cycle values ranged from 15.24 to 15.96. RT-qPCR did not detect GI.2 in any of the livers of the six hunted hares. Complete *vp60* gene sequences were determined for all three fallen hares and 10 rabbits that died of RHD in other areas of Sweden in 2016 (Genbank accession numbers MH341501–13).

**Fig. 4:**
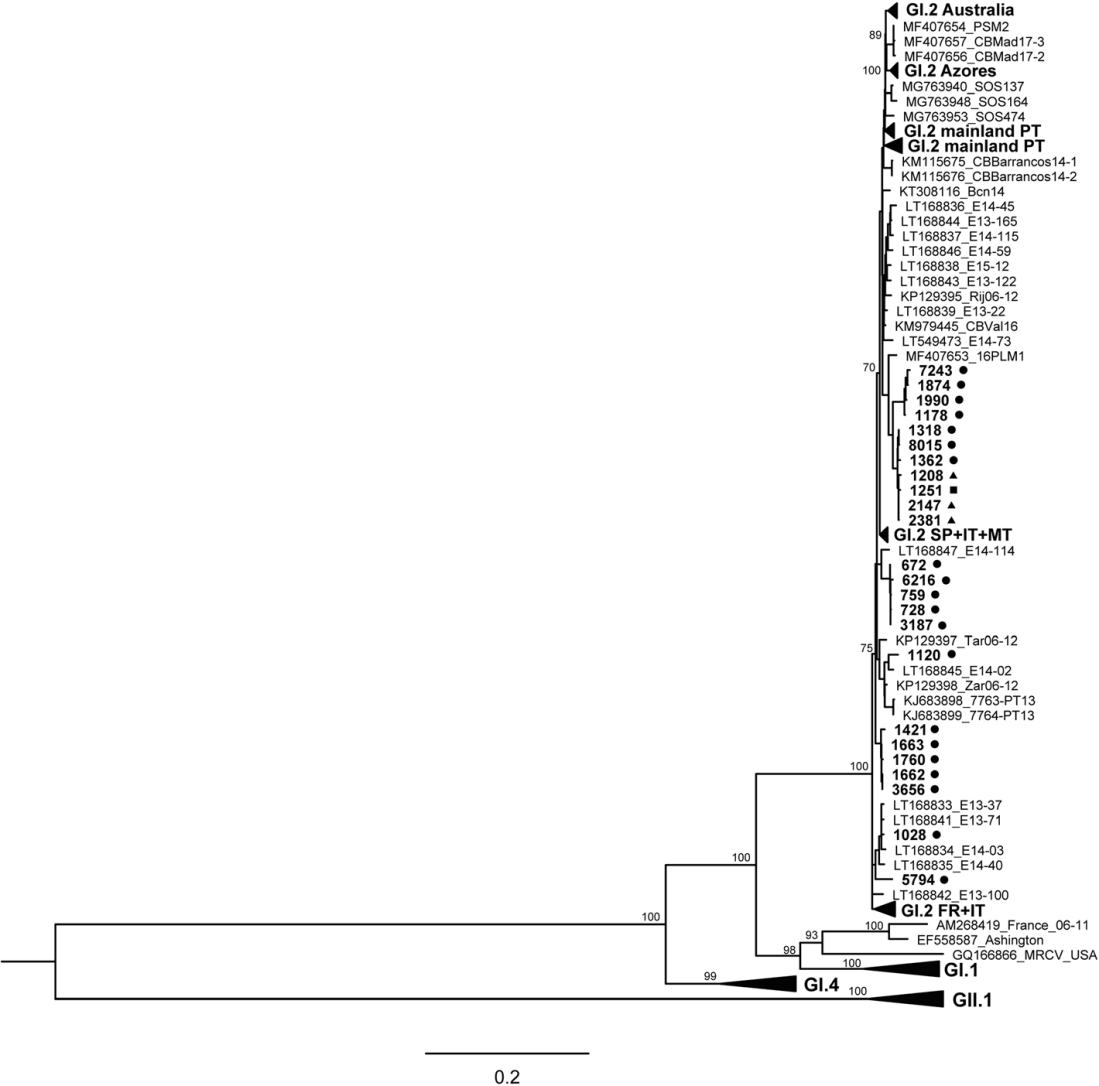
Phylogenetic analysis of complete *vp60* gene sequences of *Lagovirus europaeus* GI.2/RHDV2/b obtained from mountain hares (*Lepus timidus*) and European rabbits (*Oryctolagus cuniculus*) in Sweden in relation to other publicly available lagovirus *vp60* gene sequences. The tree was generated in MEGA6 [19] using the Maximum Likelihood method and GTR + G + I nucleotide substitution model. Bootstrap values ≤70% (of 2000 replicates) are indicated at the nodes. A complete list of GenBank accession numbers used in the analysis is provided in Additional file 1

A BLAST comparison of the *vp60* gene sequences from the three mountain hares with publicly available sequences revealed the closest identity with GI.2. More specifically, they showed a maximum identity of 98% at the nucleotide level to the 16PLM1 isolate (GenBank accession number MF407653), a non-recombinant GI.2 strain from La Palma, Canary Islands, Spain, collected in 2016 [31] (comparison performed March 20, 2018). Phylogenetic analyses grouped all three mountain hare strains together, along with other strains collected in 2016–2017 for this study (Fig. 4). Strain 1251 came from a GI.2 outbreak in rabbits in Falkenberg and was collected at the end of July 2016 on the adjacent mainland approximately 50 km away (Fig. 1). The other three strains that clustered near the hare strains (1318, 8015 and 1362) came from GI.2 outbreaks in southern mainland Sweden in July and August 2016.

## Discussion

The detection of lagoviral antigen associated with lesions of acute hepatitis, coupled with immunotyping and molecular detection of GI.2 in the liver, conclusively show that all three fallen mountain hares on Hallands Väderö died of acute viral hepatitis caused by GI.2. This is significant because, prior to this study, mountain hares were only known to be susceptible to GII.1, the pathogenic lagovirus of hares causing EBHS. The gross and microscopic pathology caused by GI.2 in mountain hares and tissue distribution of viral antigen mimics that of GII.1 infection in this species. Consistent gross lesions include a lighter coloured and friable liver, a dark red, congested and often enlarged spleen and pulmonary congestion and edema. Microscopically, periportal to massive hepatic necrosis has been described for GII.1 infections in mountain hares [32], and in this study, necrosis was classified as massive. Other lesions in these three hares that are consistent with GII.1 infections include areas of lytic necrosis, inflammation, hepatocellular fatty degeneration and mineralization of hepatocytes. Interestingly, mineralization seems to be a phenomenon of lagoviral hepatitis in hares, irrespective of the causative virus, and is not a feature commonly associated with RHD [33]. Viral antigen in the cytoplasm and nuclei of hepatocytes and cytoplasm of Kupffer cells was described both for GII.1 [31] and GI.2 in mountain hares (this study). Gross and microscopic lesions can still be considered characteristic of infection with a pathogenic lagovirus, but further analyses are required to identify the lagovirus responsible. Just as for other hare species that succumb to GI.2 infection, EBHS is no longer an adequate name for lagoviral hepatitis in mountain hares and we support the proposal by Le Gall-Reculé et al. [9] for a new name. While ‘hare hemorrhagic disease’ makes reference to the equivalent disease in rabbits (RHD), hemorrhage and disseminated intravascular coagulation (DIC) are not prominent features of pathogenic lagovirus infections in hares ([32, 34], this study). We therefore support hare lagovirus disease or hare lagoviral hepatitis as an alternate name for viral hepatitides of hares caused by different pathogenic lagoviruses.

This outbreak of GI.2 in mountain hares is also significant because it represents an expansion of GI.2 host range. GI.2 differs notably from previously described GI viruses because of its broader host range. Clinical disease from GI.1 viruses is almost exclusively restricted to the European rabbit, with the exception of GI.1 infection described in two Iberian hares (*Lepus granatensis*) [35]. In contrast, GI.2 can be pathogenic for European rabbits, but also for the Sardinian Cape hare [5], the Italian hare [6], the European brown hare [7–9] and now the mountain hare. Certain hare species are proposed to be more susceptible to GI.2 infection than others [6] and this may reflect different species-specific host factors such as glycan expression for viral attachment [36]. However, the epidemiological context may also play a role. For example, the broad and rapid dispersion of GI.2 in rabbits in France and Sardinia when compared to mainland Italy is thought to be related to the widespread presence of wild rabbits in the former areas versus sparse and patchily distributed wild rabbit populations in continental Italy [3]. Sporadic infection of European brown hares with GI.2 in mainland Italy [7] compared to large-scale outbreaks of GI.2 in European brown hares in France [9] may also reflect differences in the presence and densities of sympatric wild rabbits and thus environmental virus loads. To date, although mountain hares range throughout much of Sweden, GI.2 infection has only been detected in mountain hares on the small island of Hallands Väderö. It is unclear if all mountain hares are equally susceptible or if this particular mountain hare population was somehow predisposed to GI.2 infection. Genetic isolation, concurrent parasitic infections (found in all three dead hares and in three of four hunted hares that were examined microscopically) and the poor nutritional condition (seen in two of the three dead hares and observed in hares hunted in October 2016, B. Gunnarsson, pers. comm) may have made this population of animals more prone to GI.2 infection. Elucidation of the susceptibility of other mountain hare populations to GI.2 requires further field and/or experimental research.

Epidemiological and molecular data from the majority of previously reported GI.2 outbreaks in hares support a link to concurrent local or regional outbreaks in European rabbits [5, 6, 8, 9]. Outbreaks typically were connected in time and/or space with outbreaks in rabbits and, when data were available, the GI.2 virus in affected hares was most closely related to those strains locally circulating in rabbits [6, 9]. This suggests that while hares are susceptible to infection, they require spillover of GI.2 from the primary maintenance host, the rabbit, to initiate new outbreaks in hares. The findings presented here provide further support for this hypothesis. Although there are no rabbits on the island from which initial spillover of infection could have originated, there was a concurrent, widespread outbreak of GI.2 in wild and domestic rabbits in the southern half of Sweden in 2016 and 2017, including the adjacent mainland. The GI.2 *vp60* gene sequences from mountain hares grouped together with a sequence collected from an outbreak in rabbits at the end of July 2016 on the adjacent mainland in Falkenberg (Fig. 1), approximately 50 km away. The other *vp60* gene sequences from Sweden represent outbreaks of GI.2 in rabbits that were more distant from the outbreak on Hallands Väderö in time and/or space (Fig. 1).

Pathogenic GI viruses can be readily transmitted indirectly via contaminated material and transfer can be facilitated by insects, scavengers and people [37]. The exact mode of virus introduction onto Hallands Väderö is not known, but introduction by the 50,000 people who visit the island every year, by insects or by scavenging birds are all possibilities. Once the virus arrived on the island, GI.2 circulated for at least 4.5 months based on necropsy findings, and six months based on observations of dead hares in the field, all in the absence of rabbits. GI.2 was either introduced once to the island and then persisted for the duration of the outbreak or it was repeatedly introduced. *VP60* gene sequences of all three hares group very closely together, with only one, synonymous nucleotide substitution between the two hares found dead in 2016 and four synonymous and one non-synonymous nucleotide substitutions in the hare found dead in 2017. This lends support to a single, rather than multiple, introduction, but sequencing of the whole genome of these hares and additional rabbit samples followed by spatial and temporal analyses are needed to further substantiate this hypothesis. GI viruses are hardy and can persist in the environment for months in organic material [38]. GI.2 may have circulated within the mountain hare population during the outbreak in the absence of rabbits and/or the introduced virus may have persisted in the environment (e.g. in feces or hare carcasses), causing re-infections of hares over the period of the outbreak. Therefore, the ability of mountain hares to serve as competent maintenance hosts for GI.2 in the absence of other leporids presently is unknown and requires additional investigation.

Mountain hare populations are declining in the southern half of Sweden, coinciding geographically with where they are sympatric with brown hares [12]. Disease-mediated competition from brown hares through, for example, GII.1, has been proposed as a possible contributing factor [12]. It is now clear that mountain hares can also succumb to GI.2 infection. Wild rabbit distribution also overlaps with areas where mountain hares are in decline. The arrival of GI.2 to Sweden and subsequent large-scale epizootics in rabbits may now pose an additional threat to sympatric mountain hares. The impact of GI.2 on isolated island populations of mountain hares may be even more significant. Island populations of mountain hares are particularly vulnerable to population crashes [13]. Factors responsible for crashes such as food availability are often multifactorial and declines are often set off by a stochastic event [13]. While extreme weather and appearance of a predator have been cited as stochastic events, we propose that the sudden introduction of GI.2 could exert the same effect and trigger a decline. Continued monitoring of the hare population on Hallands Väderö is needed to assess the longer-term significance of GI.2 introduction for this population. No evidence of GI.2 infection was found in six hares hunted in January and September 2017 and, with the exception of a juvenile that died of intestinal coccidiosis, no further reports of dead hares from the island have been received to date. GI.2 has spread quickly and widely throughout Europe and beyond, and, as evidenced by this outbreak, the virus can be introduced to islands and circulate among hares in the absence of rabbits. Awareness of this potential risk coupled with actions that minimize unintended introductions of GI.2 by humans through, for example, contaminated footwear, can help mitigate exposure of other island populations of mountain hares to GI.2.

## Conclusions

We demonstrate for the first time that GI.2 can infect and cause mortality in mountain hares. These findings add a new species to the host range of GI.2. Further investigation of the mechanisms responsible for the expanded host range of GI.2 compared to GI.1 viruses could provide valuable insight into host species jumps. Lesions and virus tissue distribution of GI.2 infection in mountain hares mimic those of GII.1 infection, therefore typing of the virus must be performed to identify the lagovirus responsible for a given mortality event. The mortality event in mountain hares lasted at least 4.5 months in the absence of rabbits, but because environmental persistence and multiple introductions cannot be excluded, further research is required to determine if mountain hares are competent reservoir hosts. Phylogenetic analyses support virus introduction from a concurrent, large-scale GI.2 outbreak in rabbits on the adjacent mainland and demonstrates the relative ease with which GI.2 can spread. Finally, mountain hares represent the fourth *Lepus* species that is susceptible to GI.2. This raises the possibility that most or even all *Lepus* species may be susceptible. Not only should incidents of morbidity and mortality in other *Lepus* species sympatric with wild European rabbits or in contact with domestic rabbits be evaluated with respect to GI.2, but exposure to European rabbits should be minimized for vulnerable *Lepus* species and populations.

## Additional file

Additional file 1: GenBank accession number, sequence name and *Lagovirus europaeus* genotype (after [1]) for the *vp60* gene sequences used in the phylogenetic analysis. (XLSX 22 kb)

## Abbreviations

BLAST: Basic Local Alignment Search Tool
EBHS: European brown hare syndrome
EBHSV: European brown hare syndrome virus
MAb: Monoclonal antibody
ML: Maximum likelihood
OD: Optical density
PCR: Polymerase chain reaction),
RHD: Rabbit hemorrhagic disease
RHDV: Rabbit hemorrhagic disease virus
RT-qPCR: Reverse transcription quantitative polymerase chain reaction
